# Serum cytokines predict efficacy and toxicity, but are not useful for disease monitoring in lung cancer treated with PD-(L)1 inhibitors

**DOI:** 10.3389/fonc.2022.1010660

**Published:** 2022-10-31

**Authors:** Hannah Schindler, Fabienne Lusky, Lea Daniello, Mariam Elshiaty, Lena Gaissmaier, Karolina Benesova, Margarida Souto-Carneiro, Arlou Kristina Angeles, Florian Janke, Florian Eichhorn, Daniel Kazdal, Marc Schneider, Stephan Liersch, Sarah Klemm, Paul Schnitzler, Albrecht Stenzinger, Holger Sültmann, Michael Thomas, Petros Christopoulos

**Affiliations:** ^1^ Department of Thoracic Oncology, Thoraxklinik and National Center for Tumor Diseases (NCT) at Heidelberg University Hospital, Heidelberg, Germany; ^2^ Translational Lung Research Center Heidelberg (TLRC-H), member of the German Center of Lung Research (DZL), Heidelberg, Germany; ^3^ Department of Internal Medicine V, Hematology, Oncology and Rheumatology, Heidelberg University Hospital, Heidelberg, Germany; ^4^ Division of Cancer Genome Research (B063), German Cancer Research Center (DKFZ) and German Cancer Consortium (DKTK), Heidelberg, Germany; ^5^ Department of Thoracic Surgery, Thoraxklinik at Heidelberg University Hospital, Heidelberg, Germany; ^6^ Department of Molecular Pathology Institute of Pathology Heidelberg, Heidelberg, Germany; ^7^ Translational Research Unit, Thoraxklinik at Heidelberg University Hospital, Heidelberg, Germany; ^8^ Department of Pharmacy, Thoraxklinik and National Center for Tumor Diseases (NCT) at Heidelberg University Hospital, Heidelberg, Germany; ^9^ Center for Infectious Diseases, Virology, University Hospital Heidelberg, Heidelberg, Germany

**Keywords:** immune-checkpoint inhibitors, immunotherapy, immune-related adverse events, lung cancer, biomarker, cytokines

## Abstract

**Introduction:**

PD-(L)1 inhibitors (IO) have improved the prognosis of non-small-cell lung cancer (NSCLC), but more reliable predictors of efficacy and immune-related adverse events (irAE) are urgently needed. Cytokines are important effector molecules of the immune system, whose potential clinical utility as biomarkers remains unclear.

**Methods:**

Serum samples from patients with advanced NSCLC receiving IO either alone in the first (1L, n=46) and subsequent lines (n=50), or combined with chemotherapy (ICT, n=108) were analyzed along with age-matched healthy controls (n=15) at baseline, after 1 and 4 therapy cycles, and at disease progression (PD). Patients were stratified in rapid progressors (RP, progression-free survival [PFS] <120 days), and long-term responders (LR, PFS >200 days). Cytometric bead arrays were used for high-throughput quantification of 20 cytokines and other promising serum markers based on extensive search of the current literature.

**Results:**

Untreated NSCLC patients had increased levels of various cytokines and chemokines, like IL-6, IL-8, IL-10, CCL5, G-CSF, ICAM-1, TNF-RI and VEGF (fold change [FC]=1.4-261, p=0.026-9x10^-7^) compared to age-matched controls, many of which fell under ICT (FC=0.2-0.6, p=0.014-0.002), but not under IO monotherapy. Lower baseline levels of TNF-RI were associated with longer PFS (hazard ratio [HR]= 0.42-0.54; p=0.014-0.009) and overall survival (HR=0.28-0.34, p=0.004-0.001) after both ICT and IO monotherapy. Development of irAE was associated with higher baseline levels of several cytokines, in particular of IL-1β and angiogenin (FC=7-9, p=0.009-0.0002). In contrast, changes under treatment were very subtle, there were no serum correlates of radiologic PD, and no association between dynamic changes in cytokine concentrations and clinical outcome. No relationship was noted between the patients’ serologic CMV status and serum cytokine levels.

**Conclusions:**

Untreated NSCLC is characterized by increased blood levels of several pro-inflammatory and angiogenic effectors, which decrease under ICT. Baseline serum cytokine levels could be exploited for improved prediction of subsequent IO benefit (in particular TNF-RI) and development of irAE (*e.g.* IL-1β or angiogenin), but they are not suitable for longitudinal disease monitoring. The potential utility of IL-1/IL-1β inhibitors in the management and/or prevention of irAE in NSCLC warrants investigation.

## Introduction

Non-small-cell lung cancer (NSCLC) is the deadliest malignancy with an estimated 1.8 million deaths worldwide in 2021 (1). Most patients are diagnosed with advanced, incurable disease and a median life expectancy below two years (2). Immunotherapy (IO) with programmed death-(ligand) 1 [PD-(L)1] inhibitors, like pembrolizumab, atezolizumab and nivolumab, was a major step forward in the management of stage IV disease, facilitating long-term disease control and 5-year overall survival (OS) rates of 20-30% (3, 4). However up to 1/3 of patients do not respond, while potentially life-threatening grade III-IV immune-related adverse events (irAE) occur in approximately 10% of cases (5–7). One major unmet need is finding more reliable predictors of efficacy and toxicity to improve guidance of patient management. Tumor PD-L1 is the only currently approved biomarker (8, 9), and has also demonstrated association with the development of oligoprogression under IO (10), as well as irAE (5), but these associations are weak and complicated by considerable spatial heterogeneity of PD-L1 expression (11). Besides, the tumor mutational burden (TMB) is a pure genetic biomarker less prone to sampling errors, whose implementation has nevertheless been hampered by insufficient predictive potential and considerable technical variability (12–14). Immunologic parameters of the tumor tissue, like the emerging association of B cells and tertiary lymphoid structures with long-term IO benefit (15, 16), are an attractive alternative that directly reflects immunobiologic processes (17), but their use is limited by the scant material obtainable through small biopsies and the high procedural risk of repeated assessments. Therefore, there is increasing interest in soluble blood biomarkers that could be used to stratify patients and monitor treatment in a non-invasive manner (18). While rudimentary parameters based on routine laboratory tests, like the neutrophil-to-lymphocyte ratio (NLR, from the differential hemogram), and the advanced lung inflammation index (ALI, also incorporating the body-mass-index and serum albumin), have demonstrated predictive and prognostic utility for IO-treated NSCLC (19), a more detailed analysis of immunologic effector molecules in the blood would be expected to provide refined information and thus improve accuracy (17). Aim of this study was to systematically investigate the potential clinical utility of serum cytokines for the management of NSCLC patients treated with PD-(L)1 inhibitors.

## Materials and methods

### Patients and samples

This study included all patients with metastatic NSCLC and available serum samples, who received immunotherapy in the Thoraxklinik Heidelberg between 2012-2020, with a data cut-off on October 12^th^ 2021. PD-(L)1 inhibitors were administered either alone in the first (1L-IO) or subsequent lines (2^+^L-IO, after preceding chemotherapy), or in the 1L combined with chemotherapy (ICT). Serum samples were collected prospectively at baseline before treatment start, and longitudinally after 1 (1C) and 4 cycles (4C) of therapy, as well as at the time of disease progression (PD) (20). In order to capture more clear signals of efficacy, the focus was placed on cases with either rapid progression (RP), *i.e.* progression-free survival (PFS) < 120 days, or long-time response (LR), *i.e.* PFS > 200 days, while patients with intermediate PFS (120-200 days) were excluded from analysis. A group of age-matched healthy subjects without NSCLC was analyzed as controls.

Histological diagnosis and molecular profiling of NSCLC using combined DNA/RNA next-generation sequencing (NGS) were performed in the Institute of Pathology Heidelberg, as published (21). Patients with routinely treatable genetic alterations, like *EGFR* and *BRAF-*V600 mutations, or *ALK/ROS1/RET/NTRK* fusions, received tyrosine kinase inhibitors and were excluded from this study. The only two cases with mutations of these genes were one patient with *BRAF* p.G466E, for which no targeted therapy has been approved yet by the EMA or FDA, and one case with *MET* exon 14 skipping, for which MET inhibitors had not been approved yet at the time of the patient’s treatment, both of which received first-line chemoimmunotherapy. Clinicopathological parameters were collected from the patients’ records. The following parameters were extracted: demographics, baseline clinical and tumor characteristics including the Eastern Cooperative Oncology Group performance status (ECOG PS) and smoking status, PD-L1 tumor proportion score (TPS), results of differential blood counts, irAE characteristics, systemic anticancer treatments, date of progression, date of the last follow-up, and date of death. PD-L1 TPS was assessed using the clone SP263 (Ventana/Roche, Mannheim, Germany) and trichotomized for analysis as <1, 1–49, and ≥50%. IgG and IgM against human cytomegalovirus (CMV) was quantified using ELISA according to the manufacturer’s instructions (Euroimmun, Lübeck, Germany), and presence of either antibody class was considered to reflect positive serologic status.

PFS was defined as the time from immunotherapy start to death or progression. Overall survival (OS) was defined as the time from immunotherapy start to death or last follow-up. The progression date under immunotherapy was verified by the investigators with review of radiologic images, *i.e.* chest/abdomen CT and brain MRI-based restaging every 6–12 weeks, without formal RECIST reevaluation, as several studies have demonstrated very good agreement between real-world and RECIST-based assessments (22, 23). Diagnosis of irAE was based on standard clinicolaboratory criteria (24). This study was approved by the ethics committee of Heidelberg University (S-579/2019), and all participants gave informed consent.

### Selection of target cytokines

The cytokines in the panel were selected based on a search for original articles in PubMed on serum markers potentially associated with PD-(L)1 inhibitor efficacy in NSCLC or the development of irAE. Search terms were ((predictive biomarker[Title/Abstract]) AND ((NSCLC[Title/Abstract]) OR (lung cancer[Title/Abstract]))) with 624 results. Review articles, publications about treatments other than immunotherapy, about other tumor entities (e.g. SCLC), or about non-soluble biomarkers were excluded. After compilation of the first database, 10 additional papers were found through manual search focused on already identified potential markers. According to the published evidence and technical feasibility of multiplex measurements, the following 20 markers were selected for the current analysis: interleukin (IL)-1β, IL-2, IL-4, IL-5, IL-6, IL-8, IL-10, IL-12p70, IL-17F, interferon gamma (IFN-γ), tumor necrosis factor (TNF), intercellular adhesion molecule 1 (ICAM-1), interferon-gamma induced protein 10 (IP-10), vascular endothelial growth factor (VEGF), angiogenin, soluble CD40 ligand (sCD40L), granulocyte-colony stimulating factor (G-CSF), CC motif chemokine ligand 5 (CCL5), granzyme a, and soluble TNF-receptor I (TNF-RI). The results of the literature search and the rationale for selection of analyzed markers are shown in **Supplementary Table 1**.

### Sample processing and cytokine testing

Blood was collected in lithium-heparin tubes, centrifuged at 2000 g for 10 minutes within 1 h from venipuncture, followed by removal, aliquoting and storage of serum at -80°C. For the quantification of cytokines, aliquots were thawed on ice and measured using cytometric bead arrays (CBA) according to the manufacturer’s instructions (Beckton Dickinson, Heidelberg, Germany) with standard (S) or enhanced (E) sensitivity kits, as appropriate. The limit of detection and range of assays used in this study is shown in **Supplementary Table 2**. In brief, each sample was centrifuged at 12000 g for 2 min at 4°C, and the supernatant transferred into a new tube and diluted 1:3 (S) or 1:4 (E) to a total volume of 50 µl. Capture beads were diluted 1:20 (for E kits, followed by a single wash step using 1 ml of wash buffer at 200g for 5 minutes) or 1:50 (for S kits), added to the samples (E: 20 µl; S: 50 µl), and incubated at room temperature (E: 2 h; S: 1 h). Next, the phycoerythrin (PE) detection reagent was added (E: 20 µl diluted 1:20; E: 50 µl diluted 1:50), samples were incubated for 2 h at room temperature in the dark, for E kits the second component of the detection diluent 1:10 diluted was added, and all samples underwent a final washing step with resuspension in 200 μl of wash buffer for measurement. Standard curves were generated by processing the lyophilized standards provided with the kits in a similar way as the patients’ samples. For sample acquisition, an LSR-Fortessa Flow Cytometer (Beckton Dickinson, Heidelberg, Germany) was used. Cytokine concentrations were calculated from the raw CBA data using the Fcap Array™ version 3.0 software (Soft Flow, Pecs, Hungary).

### Statistical analysis

Statistical comparisons between patient groups (*e.g.* RP *vs.* LR) were performed using Wilcoxon tests, while paired Wilcoxon tests were used to analyze different time-points of the same patients (*e.g.* baseline *vs.* 4C). Fold change (FC) was calculated trough division of mean values. Survival was analyzed according to Kaplan-Meier and compared between groups using log-rank tests, after determining the optimal cut-off based on ROC and Youden-index analysis. The association with various parameters with survival was explored using Cox regression. The correlation between clinicopathological variables and serum markers was analyzed according to Spearman, while correlations classified as very weak (|r|<0.2), weak (|r|=0.2-0.3), moderate (|r|=0.3-0.5), and strong (|r|=0.5-0.7). Multiple testing correction was performed according to Benjamini- Hochberg. Statistical calculations were performed with SPSS version 28 (IBM Corp., Armonk, NY, USA) and R version 4.2.1 (www.R-project.org). Two-tailed p-values lower than 0.05 and with false discovery rate (FDR) lower 0.1 were considered significant.

## Results

### Patient characteristics

**Figure 1:**
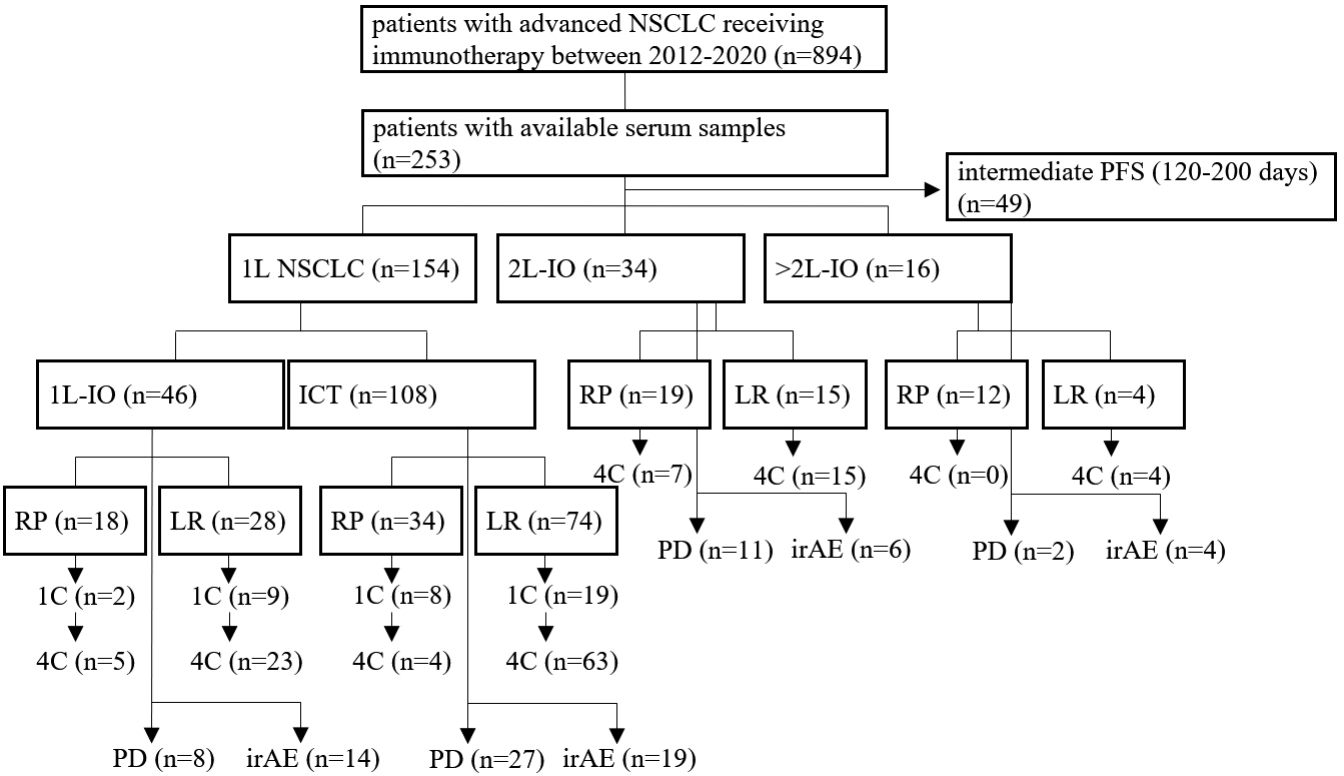
Flowchart of study patients. A total of 894 patients with metastatic NSCLC from 2012-2020 was treated with immunotherapy (IO), *i.e.* either PD-(L)1 inhibitors alone or in combination with chemotherapy (ICT), of which serum samples were available for 253. This study focused on patients with either rapid progression (RP, *i.e.* within 120 days from immunotherapy start), or long-term benefit (LR, *i.e.* progression-free survival > 200 days), as explained in the *Materials and Methods*. Treatment was either in the first (1L) or in the second and subsequent lines (2+L IO). Samples were collected at baseline, after 1 cycle of treatment (1C), after 4 cycles of treatment (4C), or at the time of disease progression (PD), as possible. Some patients developed immune-related adverse-events (irAE), which were analyzed separately.

A total of 204 patients with metastatic NSCLC who received PD-(L)1 inhibitors in the first or subsequent lines could be included in the study (**Figure 1**). An overview of characteristics for study patients is given in **Table 1**, while more details about treatment type and stratification based on IO efficacy are provided in **Supplementary Table 3**. Mean age was 65 years (range 37-87) for first-line patients (n=154), 63 years (range 48-78) for patients in subsequent lines (n=50, **Table 1**), and 66 years (range 58-81) for age-matched healthy donors (n=15).

**Table 1:** Patient characteristics.

	All NSCLC patients(n=894)	1L NSCLC(n=154)		≥2L NSCLC(n=50)	
**Age (mean, range)**	65 (25-91)	65 (37-87)		63 (48-78)	
**Sex (n, %)**					
** Female** ** Male**	358 (40%) 536 (60%)	51 (33%) 103 (67%)		24 (49%) 26 (51%)	
**Smokers (n, %)**					
** never** ** former** ** current**	80 (9%) 456 (51%) 358 (40%)	15 (10%) 76 (49%) 63 (41%)		1 (4%) 28 (55%) 21 (41%)	
**ECOG (n, %)^1^**					
** 0** ** 1** ** ≥2**	411 (46%) 465 (52%) 18 (2%)	67 (44%) 84 (54%) 3 (2%)		19 (38%) 25 (50%) 6 (12%)	
**PD-L1 (n, %)**					
** <1** ** 1-49** ** ≥50**	178 (20%) 367 (41%) 349 (39%)	34 (22%) 56 (36%) 64 (42%)		13 (27%) 28 (55%) 9 (18%)	
**Histology (n, %)**					
** ADC** ** SCC** ** Other NSCLC**	626 (70%) 202 (23%) 66 (7%)	109 (70,5%) 28 (18,6%) 17 (10,9%)		33 (65%) 12 (24%) 5 (11%)	
**Immunotherapy^1^**					
** anti-PD-1** ** anti- PD-L1**	803 (90%) 91 (10%)	151 (98%) 3 (2%)		35 (71%) 15 (29%)	
**irAE (n, %)**					
** Yes** ** No**	198 (22%) 696 (78%)	33 (21%) 121 (79%)		11 (24%) 39 (76%)	

1L NSCLC: patients receiving (chemo-)immunotherapy in the first line; ≥2L NSCLC patients receiving PD-(L)1 inhibitors as monotherapy in the second-or-subsequent lines; ECOG, Eastern Cooperative Oncology Group; PD-L1, programmed cell death protein ligand 1; ADC, adenocarcinoma; SCC, squamous-cell carcinoma; NSCLC, non-small-cell lung cancer; irAE, immune-related adverse events; 1L, first line.

^1^PD-1-inhibitors: nivolumab, pembrolizumab; PD-L1-inhibitors: atezolizumab, durvalumab.

Further details, like type of treatment, and stratification according to clinical benefit from immunotherapy, are given in **Supplementary Table 3**.

### Serum cytokine profile of advanced NSCLC at baseline

**Figure 2:**
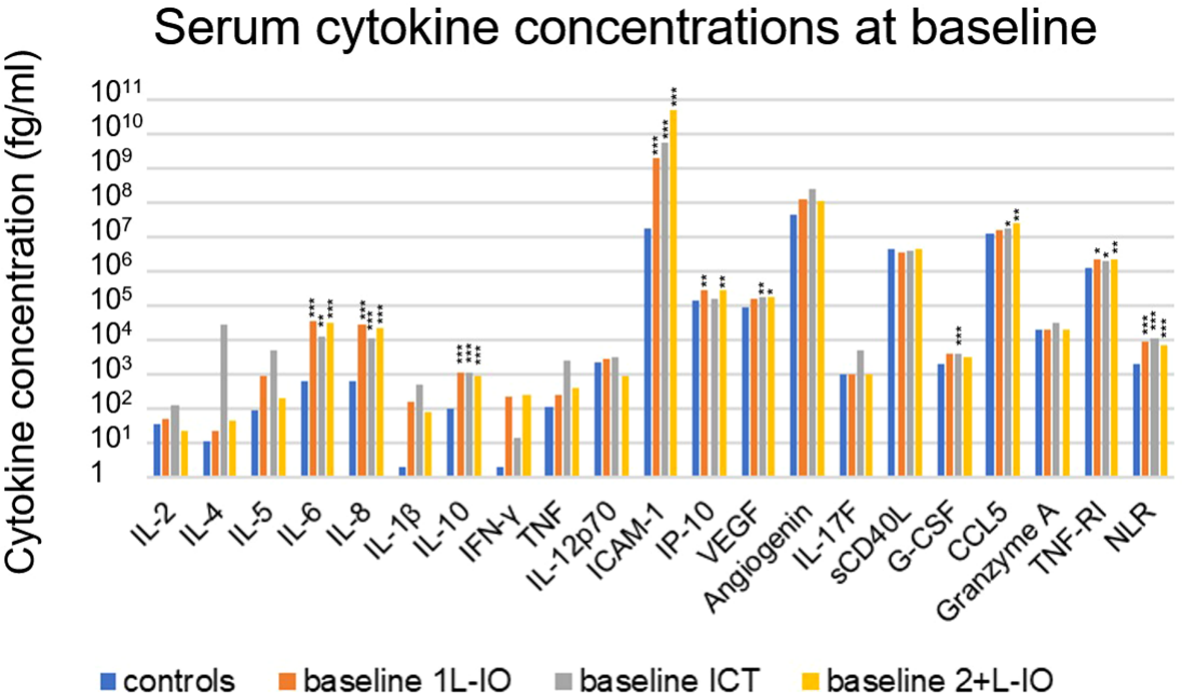
Cytokine levels before immunotherapy start in newly diagnosed and chemotherapy-pretreated NSCLC compared to age-matched healthy control donors. Shown is the mean concentration of each cytokine in first-line patients at baseline, as well as in age-matched healthy controls. An overview of significant results and the explanation of abbreviations is shown in **Table 2**. In newly diagnosed patients IL-6 (FC=31, p=0.0002), IL-8 (FC=25, p=4x10^-8^), IL-10 (FC=11, p=0.0002), CCL5 (FC=1.4, p=0.026), G-CSF (FC=5, p=0.004), ICAM-1 (FC=261, p=9x10^-7^), TNF-RI (FC=1.6, p=0.021), VEGF (FC=2, p=0.007) and NLR (FC=5, p=10^-9^) were elevated. In pretreated patients IL-6 (FC=49, p=5x10^-6^), IL-8 (FC=34, p=7x10^-8^), IL-10 (FC=9 p=4x10^-6^), IP-10 (FC=2, p=0.006), CCL5 (FC=1.9, p=0.001), ICAM-1 (FC=2839, p=9x10^-7^), TNF-RI (FC=1.7, p=0.001), VEGF (FC=2, p=0.017) and NLR (FC=4, p=4x10^-7^) were elevated as well; *=p<0.05; **=p<0.01; ***=p<0.001.

Several cytokines were significantly increased in the serum of untreated patients compared to age-matched healthy controls, *i.e.* IL-6, IL-8, IL-10, CCL5, G-CSF, ICAM-1, TNF-RI and VEGF (fold-change [FC]=1.4-261, p=0.026-9x10^-7^, **Table 2** and **Figure 2**). Besides, chemotherapy-pretreated 2^+^L patients before start of immunotherapy in later lines showed similar changes, with significantly increased serum concentrations of IL-6, IL-8, IL-10, IP-10, CCL5, ICAM-1, TNF-RI and VEGF compared to the controls (FC= 1.7-2839; p=0.017–7x10^-8^), but mixed changes compared to untreated, newly diagnosed patients: IL-6, IP-10, CCL5 and ICAM-1 were significantly increased (FC= 1.4-11; p=0.011–0.0007), while IL-2 and G-CSF were decreased (FC= 0.2-0.3; p=0.005–0.008, **Table 2** and **Figure 2**). Of note, the baseline NLR was increased in all patient subgroups compared to controls, but decreased in 2^+^L compared to untreated patients (**Table 2**).

**Table 2:** Profile of blood markers in study patients and healthy controls.

	Patients at baseline vs. ctrl (n=15)				Levels under treatment									LR vs. RP ^1^				irAE
Marker	untreated			pretreated	2^+^L-IO vs. untreated(n=204)	ICT (vs. baseline)			1L-IO (vs. baseline)			2^+^L-IO (vs. baseline)		baseline ^1^	under treatment			baseline vs. ctrl ^1^
	all 1L(n=154)	ICT(n=108)	1L-IO(n=46)	2^+^L-IO(n=50)		C1 vs. bl(n=27)	C4 vs bl(n=67)	PD vs. C4(n=10)	C1(n=11)	C4(n=28)	PD vs. C4(n=6)	C4(n=26)	PD vs. C4(n=9)		C1 ^1^	C4 ^1^	PD ^1^	
**IL-1β**	FC>10 p=0.055	FC>10 p=0.028	FC>10 p=0.202	FC>10 p=0.157	FC=0.2 p=0.316	FC=1 p=1	FC=1.2 p=0.959	FC<0.1 p=0.18	FC<0.1 p=0.317	FC=14 p=0.593	FC=1 p=1	FC=0.1 p=0.655	FC=1 p=1	FC=0.8-3 p>0.5	FC=1 p>0.5	FC=1.4-10 p>0.07	FC=0.1-10 p>0.3	FC=7 p=0.009^$^
**IL-2**	FC=3 p=0.328	FC=4 p=0.397	FC=1.4 p=0.265	FC=0.7 p=0.024	FC=0.2 p=0.005	FC=0.5 p=0.18	FC=0.8 p=0.674	FC<0.1 p=0.18	FC=0.3 p=0.317	FC=27 p=0.317	FC=1 p=1	FC=0.7 p=0.686	FC<0.1 p=0.317	FC=0.8-9 p=>0.2	FC>10 p>0.35	FC=0.5-10 p>0.6	FC<0.1-1 p>0.4	FC=0.1-4 p>0.1
**IL-4**	FC=148 p=0.128	FC=225 p=0.104	FC=1.8 p=0.241	FC=4 p=0.121	FC<0.1 p=0.804	FC=0.1 p=0.109	FC<0.1 p=0.019	FC<0.1 p=0.109	FC=0.6 p=0.715	FC=7 p=0.786	FC=0.1 p=0.285	FC=1.3 p=0.753	FC=0.3 p=0.18	FC=0.5-49 p>0.5	FC>10 p>0.40	FC=0.2-10 p>0.1	FC=0.7-10 p>0.3	FC=1.7-88 p>0.06
**IL-5**	FC=7 p=0.052	FC=6 p=0.052	FC=10 p=0.073	FC=2 p=0.32	FC=0.3 p=0.180	FC<0.1 p=0.08	FC=1.1 p=0.701	FC<0.1 p=0.285	FC=0.3 p=0.273	FC=1.4 p=0.61	FC<0.1 p=0.317	FC=2 p=0.686	FC=1 p=1	FC=3-213 p=0.03-0.5	FC<0.1-10 p>0.4	FC=6-10 p>0.19	FC<0.1-1 p>0.8	FC=4 p=0.011^$^ FC=17 p=0.003^#^
**IL-6**	FC=31 p=0.0002	FC=20 p=0.004	FC=56 p=10^-6^	FC=49 p=5E-06	FC=1.6 p=0.0007	FC=0.2 p=0.002	FC=6 p=0.695	FC=0.6 p=0.508	FC=0.8 p=0.575	FC=0.5 p=0.043	FC=0.4 p=0.5	FC=0.5 p=0.086	FC=2 p=0.327	FC=0.7 p=0.005^$^	FC=0.1-0.8 p>0.4	FC<0.1-2 p>0.11	FC=0.1-12 p>0.11	FC=0.4-1 p>0.2
**IL-8**	FC=25 p=4E-08	FC=17 p=1E-07	FC=42 p=3x10^-7^	FC=34 p=7x10^-8^	FC=1.4 p=0.052	FC=0.3 p=0.003	FC=0.7 p=0.025	FC=0.5 p=0.799	FC=0.5 p=0.062	FC=0.4 p=0.179	FC=0.5 p=0.345	FC=0.7 p=0.238	FC=3 p=0.314	FC=0.7 p=0.02-0.6	FC=1.7-1.9 p>0.16	FC=0.5-1.4 p>0.3	FC=0.6-4 p>0.13	FC=1.6 p=0.028 ^$^
**IL-10**	FC=11 p=0.0002	FC=11 p=3E-04	FC=11 p=4x10^-4^	FC=9 p=4E-06	FC=0.3 p=0.231	FC=1.3 p=0.314	FC=1.7 p=0.215	FC<0.1 p=0.08	FC=3 p=1	FC=1.3 p=0.913	FC=0.5 p=0.273	FC=1.3 p=0.753	FC=0.7 p=0.893	FC=1-2 p>0.4	FC=0.7-10 p>0.2	FC=0.4-7 p>0.2	FC<0.1-10 p=0.03-0.7	FC=3 p=0.004 ^$^
**IP-10**	FC=1.4 p=0.497	FC=1.1 p=0.688	FC=2 p=0.006	FC=2 p=0.006	FC=1.4 p=0.011	FC=1.7 p=0.349	FC=1.7 p=0.001	FC=0.9 p=0.575	FC=3 p=0.062	FC=1.4 p=0.031	FC=0.6 p=0.075	FC=0.9 p=0.159	FC=2 p=0.314	FC=0.7-0.8 p=0.04-0.4	FC=0.2-1.5 p>0.1	FC=0.3-0.7 p>0.1	FC=0.6-1.4 p>0.1	FC=0.6-1.4 p>0.1
**IL-12p70**	FC=1.2 p=0.954	FC=1.3 p=0.689	FC=1.2 p=0.519	FC=0.4 p=0.397	FC=0.3 p=0.253	FC=0.5 p=0.012	FC=0.8 p=0.638	FC=0.2 p=0.144	FC=0.3 p=0.465	FC=1.6 p=0.893	FC=1 p=1	FC=7 p=0.593	FC>10 p=0.18	FC=0.4 p>0.05	FC>10 p>0.2	FC=0.6->10 p>0.3	FC=1-17 p>0.4	FC=4 p=0.019^$^ FC=9 p=0.014^#^
**IL-17F**	FC>10 p=0.529	FC>10 p=0.515	FC>10 p=0.421	FC>10 p=0.584	FC=0.01 p=0.805	FC=1 p=1	FC<0.1 p=0.317	FC=1 p=1	FC=1 p=1	FC=46 p=0.285	FC=1 p=1	FC<0.1 p=0.317	FC=1 p=1	FC>10 p>0.19	FC=1 p=1	FC=1->10 p>0.5	FC=1 p=1	FC<0.1-10 p=0.5-0.4
**CCL5**	FC=1.4 p=0.026	FC=1.4 p=0.016	FC=1.2 p=0.132	FC=1.9 p=0.001	FC=1.4 p=0.002	FC=1.2 p=0.088	FC=1.05 p=0.358	FC=0.9 p=0.646	FC=0.9 p=0.79	FC=1.1 p=0.767	FC=0.7 p=0.463	FC=0.7 p=0.012	FC=1.1 p=0.374	FC=0.8-1.1 p>0.17	FC=0.9-1.1 p>0.4	FC=0.7-0.8 p>0.07	FC=8-1.2 p>0.3	FC=0.7-0.9 p>0.08
**sCD40L**	FC=0.9 p=0.141	FC=0.9 p=0.228	FC=0.8 p=0.092	FC=1 p=0.213	FC=1.1 p=0.859	FC=1.1 p=0.829	FC=1 p=0.336	FC=1.1 p=0.114	FC=0.5 p=0.003	FC=1 p=0.946	FC=0.8 p=0.753	FC=1 p=0.581	FC=1 p=0.767	FC=0.7-1.1 p=0.05-0.7	FC=0.6-1.9 p>0.1	FC=0.5-1.1 p>0.1	FC=1.1-1.3 p>0.1	FC=0.5-1.2 p=0.01-0.6
**G-CSF**	FC=5 p=0.004	FC=6 p=10^-4^	FC=2 p=0.564	FC=1.6 p=0.297	FC=0.3 p=0.008	FC=2 p=0.001	FC=0.4 p=0.733	FC=0.6 p=0.285	FC=0.9 p=0.328	FC=1.3 p=0.889	FC=0.2 p=0.116	FC=1 p=0.689	FC=1.7 p=0.213	FC=0.4-1.5 p>0.3	FC=0.7-1.7 p>0.2	FC=0.5-0.8 p>0.2	FC=0.3-1.7 p=0.05-0.4	FC=0.7 p=0.027^$^
**Granzyme A**	FC=1.5 p=0.903	FC=1.7 p=0.567	FC=1 p=0.384	FC=1 p=0.72	FC=0.7 p=0.691	FC=0.8 p=0.049	FC=0.7 p=0.797	FC=0.9 p=0.799	FC=1.5 p=0.594	FC=1 p=0.716	FC=0.2 p=0.249	FC=0.5 p=0.551	FC=1.4 p=0.314	FC=0.5-2 p>0.1	FC=0.8-1.9 p>0.08	FC=0.9-2 p>0.1	FC=0.2-0.6 p>0.1	FC=1.3 p=0.003^#^
**ICAM-1**	FC=261 p=9x10-^7^	FC=326 p=4x10^-7^	FC=110 p=0.0001	FC=2839 p=9x10^-7^	FC=11 p=0.011	FC=0.3 p=0.203	FC=0.3 p=0.0005	FC=0.8 p=0.59	FC=0.6 p=0.003	FC=0.7 p=0.317	FC=0.2 p=0.917	FC=8 p=0.0004	FC=0.2 p=0.008	FC<0.1-2 p>0.1	FC=0.2-1.8 p>0.03	FC<0.1-24 p>0.15	FC=2-9 p>0.01	FC=0.1-0.5 p>0.2
**IFN-γ**	FC>10 p=0.603	FC>10 p=0.65	FC>10 p=0.382	FC>10 p=0.494	FC=3 p=0.641	FC=1 p=1	FC=1 p=1	FC=1 p=1	FC=1 p=1	FC=4 p=0.317	FC=1 p=1	FC=1 p=1	FC=1 p=1	FC>10 p>0.2	FC=1 p=1	FC=1->10 p>0.6	FC=1 p=1	FC<0.1-10 p>0.1
**TNF**	FC=14 p=0.829	FC=20 p=0.995	FC=2 p=0.587	FC=3 p=0.869	FC=0.2 p=0.488	FC=0.6 p=0.136	FC=0.5 p=0.039	FC=0.4 p=0.345	FC=0.5 p=0.779	FC=23 p=0.695	FC=9 p=0.109	FC=1.2 p=0.79	FC=1.2 p=0.5	FC=0.8.8 p>0.5	FC=1.2-10 p>0.09	FC=0.2-20 p>0.19	FC=0.3-4 p=0.044-1	FC=0.1-6 p=0.02-0.9
**TNF-RI**	FC=1.6 p=0.021	FC=1.5 p=0.03	FC=1.8 p=0.021	FC=1.7 p=0.001	FC=1.1 p=0.095	FC=0.9 p=0.136	FC=0.9 p=0.153	FC=1.2 p=0.074	FC=0.9 p=0.286	FC=0.8 p=0.084	FC=1 p=0.753	FC=0.9 p=0.101	FC=1.2 p=0.374	FC=0.8 p=0.006^$^ FC=0.5 p=0.003*	FC=0.7-1 p=0.124-1	FC=0.3-0.8 p>0.024	FC=0.4-0.9 p>0.025	FC=0.7-1.1 p>0.062
**Angiogenin**	FC=4 p=0.658	FC=5 p=0.391	FC=3 p=0.582	FC=2 p=0.513	FC=0.5 p=0.45	FC=0.6 p=0.014	FC=0.3 p=7E-05	FC=2 p=0.575	FC=0.7 p=0.006	FC=3 p=0.838	FC<0.1 p=0.463	FC=0.3 p=1E-05	FC=1.1 p=0.859	FC=0.6-3 p>0.031	FC=1-1.5 p>0.4	FC=1.7-31 p>0.018	FC=0.3-1.4 p>0.1	FC=9 p=0.0002 ^$^
**VEGF**	FC=2 p=0.007	FC=2 p=0.003	FC=1.9 p=0.056	FC=2 p=0.017	FC=1.1 p=0.954	FC=1.2 p=0.097	FC=1 p=0.779	FC=1.1 p=0.959	FC=0.8 p=0.182	FC=0.8 p=0.043	FC=1.1 p=0.345	FC=0.7 p=0.101	FC=1.6 p=0.021	FC=0.8-1 p>0.1	FC=0.8 p>0.1	FC=0.3-1 p>0.08	FC=0.7-1.5 p>0.4	FC=0.7-1.3 p>0.2
**NLR**	FC=5 p=10^-9^	FC=6 p=10^-9^	FC=5 p=2x10^-7^	FC=4 p=4x10^-7^	FC=0.7 p=0.001	FC=0.7 p=0.019	FC=0.6 p=2x10^-5^	FC=0.9 p=0.388	FC=1.5 p=0.721	FC=0.6 p=0.116	FC=0.9 p=0.465	FC=1.1 p=0.683	FC=1 p=0.594	FC=0.8 p=0.024^*^	FC=0.5-0.9 p>0.1	FC=0.5 p=0.029^*^ FC=0.6 p=0.022^#^	FC=0.6-1.4 p=0.2-1	FC=0.6-1.1 p>0.1

IL, interleukin; IFN-γ, interferon gamma; TNF, tumour necrosis factor; ICAM-1, intercellular adhesion molecule 1; IP-10, interferon-gamma induced protein 10; VEGF, vascular endothelial growth factor; sCD40L, soluble CD40 ligand; G-CSF, granulocyte-colony stimulating factor; CCL5, CC motif chemokine ligand 5; TNF-RI, soluble TNF-receptor I; ICT, immunochemotherapy; 1L-IO, patients receiving PD-(L)1 inhibitors as monotherapy in the first line; ctrl, age-matched healthy controls; 2^+^L-IO, patients receiving PD-(L)1 inhibitors as monotherapy in the second-or-subsequent lines; BL, baseline; C1, sample after 1 cycle of treatment; C4, sample after 4 cycles of treatment; FC, fold change; NLR, neutrophil-to-lymphocyte ratio; ^$^, for the ICT subgroup; *, for the 1L-IO subgroup; ^#^,for the 2+L-IO subgroup; for detailed results please see **Supplementary Table 4**.

Non-parametric statistical comparisons (please see Methods). Comparisons significant after B-H correction (FDR <0.1) are highlighted in green. Results with p<0.05, but FDR >0.1 are highlighted in grey.

^1^for the group sizes please see **Figure 1**.

### Serum cytokine changes under treatment with PD-(L)1 inhibitors

**Figure 3:**
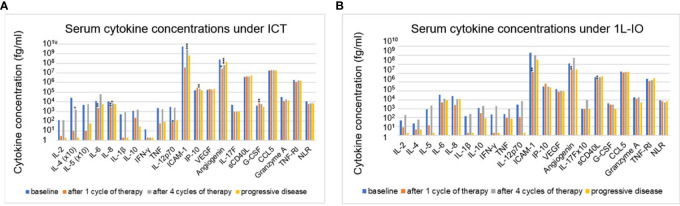
Changes of serum cytokines levels under immunotherapy in NSCLC patients compared to baseline values. **(A)** for patients receiving first-line immunochemotherapy (ICT): baseline (BL), after 1 cycle of treatment (C1), after 4 cycles of treatment (C4), at the time of disease progression (PD). **(B)** for patients receiving first-line PD-(L)1 monotherapy (1L-IO): baseline (BL), after 1 cycle of treatment (C1), after 4 cycles of treatment (C4), at the time of disease progression (PD). Shown is the mean concentration of each cytokine in the respective patients. An overview of significant results and the explanation of abbreviations is shown in **Table 2**; *=p<0.05; **=p<0.01; ***=p<0.001.

In contrast to prominent aberrations at the time of immunotherapy start, serum cytokine changes under treatment with PD-(L)1 inhibitors were subtle (**Figures 3A, B**). Most consistent was a decrease in angiogenin after 1 cycle in the first line (FC=0.6-0.7, p=0.014-0.006) or after 4 cycles in later lines (FC=0.3, p=10^-5^). Other changes were either inconsistent, *i.e.* ICAM-1 dropped under IO-monotherapy in the first line, but increased under treatment in later lines; sCD40L was lower under treatment in the first, but not in subsequent lines; CCL5 was decreased under treatment in subsequent, but not in the first line; or concerned chemoimmunotherapy only, but not PD-(L)1 monotherapy, *i.e.* decreases of IL-6, IL-8, IL-12p70, and increases in IP-10 and G-CSF (**Figures 3A, B**). The NLR also decreased under treatment, but only in patients receiving ICT (**Figures 3A, B**). No significant changes were observed at the time of disease progression compared to the levels after 4 treatment cycles (**Figures 3A, B**).

### Serum cytokine changes associated with survival

**Figure 4:**
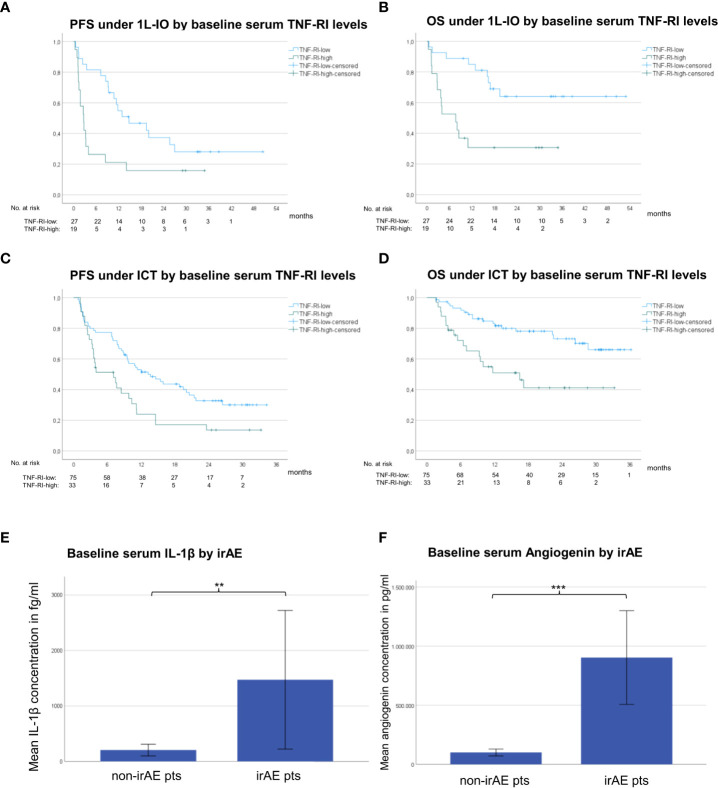
Serum cytokines associated with immunotherapy efficacy and toxicity in NSCLC. **(A)** The median progression-free survival (PFS) under PD-(L)1 monotherapy for patients with low TNF-RI at diagnosis (<2139.7 pg/ml, please see **Supplementary Figure 1**) was 442 days *vs.* 80 days for patients with high TNF-RI. **(B)** The median overall survival (OS) under PD-(L)1 monotherapy for patients with low TNF-RI at diagnosis (<2139.7 pg/ml) was not reached *vs.* 229 days for patients with high TNF-RI. **(C)** The median PFS under immunochemotherapy (ICT) for patients with low TNF-RI at diagnosis (<2139.73 pg/ml, please see **Supplementary Figure 1**) was 409 days *vs.* 212 days for patients with high TNF-RI. **(D)** The median OS under ICT for patients with low TNF-RI at diagnosis (<2139.73 pg/ml) was not reached *vs.* 493 days for patients with high TNF-RI. **(E)** The mean baseline serum IL-1β concentration for patients with metastatic NSCLC receiving first-line ICT who subsequently developed irAE was 1472 fg/ml vs. 206 fg/ml for patients without irAE; **=p<0.01. Error bars indicated standard error of the mean. **(F)** The mean baseline serum angiogenin concentration for patients with metastatic NSCLC receiving first-line ICT who subsequently developed irAE was 903,463 pg/ml vs. 100,254 pg/ml for patients without irAE; ***=p<0.001. Error bars indicated standard error of the mean.

For both first-line PD-(L)1 inhibitor monotherapy and ICT cohorts, LR patients had significantly lower TNF-RI levels at baseline compared to RP patients (FC=0.5-0.8, p=0.006-0.003, **Table 2**). The respective TNF-RI cut-off was 2139.7 pg/ml, as determined by receiver operating characteristic (ROC) curve and Youden index analysis (**Supplementary Figure 1**). Patients with low TNF-RI baseline levels receiving PD-(L)1 monotherapy showed longer PFS (442 *vs.* 80 days in median, hazard ratio [HR] = 0.42, p=0.014) and OS (not reached *vs.* 229 days, HR = 0.28, p=0.004) compared to patients with high TNF-RI levels, **Figures 4A, B**). Besides, patients with low TNF-RI baseline levels receiving ICT showed longer PFS (409 *vs.* 212 days in median, hazard ratio [HR] = 0.53, p=0.009) and OS (not reached *vs.* 493 days, HR = 0.34, p=0.001) compared to patients with high TNF-RI levels, **Figures 4C, D**). Additionally, lower IL-6 levels were also significantly linked with IO efficacy for patients receiving ICT (**Table 2**), but other associations did not exceed the FDR<0.1 threshold (**Supplementary Table 4**). Patients with lower IL-6 levels at baseline receiving ICT showed longer PFS (436 *vs.* 212 days in median, HR = 0.50, p=0.003) and OS (not reached *vs.* 514 days in median, HR = 0.29, p=0.0003) than patients with higher IL-6 levels. No differences in cytokine levels under treatment and during disease progression were observed between LR and RP after correction for multiple testing (**Table 2** and **Supplementary Table 4**). The NLR at baseline and after 4 cycles of treatment was associated with LR in patients receiving PD-(L)1 monotherapy, but not in patients receiving ICT (**Table 2**).

### Baseline serum cytokines levels associated with the development of irAE

Several cytokine abnormalities were evident in baseline samples of patients receiving ICT, who subsequently developed irAE (**Figures 4E, F**), *i.e.* in order of decreasing degree of association: elevated baseline levels of angiogenin (FC=9, p=0.0002), IL-1β (FC=7, p=0.009), IL-5 (FC=4, p=0.011), IL-12p70 (FC=4, p=0.019), IL-10 (FC=3, p=0.004), and IL-8 (FC=1.6, p=0.0285), as well as reduced baseline levels of G-CSF (FC=0.7, p=0.007). In addition, patients developing irAE under PD-(L)1 monotherapy in later lines showed higher baseline levels of IL-5 (FC=17, p=0.003), IL-12p70 (FC=9, p=0.014), and granzyme A (FC=1.3, p=0.003, **Table 2**). The characteristics of irAE are shown in **Supplementary Table 5**. No significant changes of the aforementioned cytokines were noted according to the severity or irAE (grade 1/2 *vs.* 3/4) or the use of steroids or not (data not shown).

### Serum cytokine levels associated with clinical characteristics

Several cytokines were associated with each other, most notably IL-1β, IL-2, IL-4, IL-5, IL-6, IL-8, IL-10, IL-12p70, ICAM-1, TNF and angiogenin formed a cluster with multiple (>1) moderate (r>0.3) significant correlations with each other (**Table 3**). Notable was also a weak, but significant (r=0.28, p=5x10^-9^) correlation between TNF-RI and IL-6, the two cytokines more prominently linked to immunotherapy efficacy, as described in the previous section *Serum cytokine changes associated with survival*.

**Table 3:** Overview of the associations between blood cytokine levels and patient characteristics.

	sCD40L	CCL5	VEGF	G-CSF	NLR	IL-17F	IP-10	TNF-RI	IFN-γ	Granzyme A	TNF	IL-12p70	IL-2	IL-4	IL-1β	ICAM- 1	Angiogenin	IL-6	IL-5	IL-10	IL-8
**IL-8**	r=-0.052 p=0.284	r=0.092 p=0.059	r=0.165 p=0.001	r=-0.016 p=0.737	r=0.086 p=0.08	r=0.009 p=0.857	r=0.203 p=3E-05	r=0.296 p=6E-10	r=0.141 p=0.009	r=0.23 p=2E-06	r=0.129 p=0.017	r=0.189 p=4x10^-4^	r=0.257 p=1x10^-6^	r=0.237 p=9x10^-6^	r=0.270 p=4x10^-7^	r=0.337 p=10^-12^	r=0.359 p=2x10^-14^	r=0.617 p=10^-45^	r=0.444 p=5x10^-18^	r=0.6 p=5x10^-35^	
**IL-10**	r=-0.025 p=0.648	r=0.045 p=0.401	r=0.13 p=0.016	r=0.197 p=2E-04	r=0.171 p=0.002	r=0.022 p=0.688	r=0.121 p=0.025	r=0.246 p=4x10^-6^	r=0.176 p=0.001	r=0.237 p=9x10^-6^	r=0.211 p=8x10^-5^	r=0.291 p=4x10^-8^	r=0.31 p=4x10^-9^	r=0.354 p=10^-11^	r=0.386 p=1x10^-13^	r=0.395 p=3x10^-14^	r=0.393 p=4x10^-14^	r=0.456 p=4x10^-19^	r=0.587 p=3x10^-33^		
**IL-5**	r=-0.069 p=0.203	r=-0.142 p=0.009	r=0.084 p=0.119	r=0.099 p=0.066	r=0.034 p=0.534	r=0.121 p=0.024	r=0.092 p=0.09	r=0.04 p=0.461	r=0.223 p=3x10^-5^	r=0.324 p=8x10^-10^	r=0.189 p=4x10^-4^	r=0.453 p=9x10^-19^	r=0.277 p=2x10^-7^	r=0.306 p=7x10^-9^	r=0.395 p=3x10^-14^	r=0.263 p=8x10^-7^	r=0.408 p=3x10^-15^	r=0.354 p=10^-11^			
**IL-6**	r=0.008 p=0.863	r=-0.026 p=0.601	r=0.145 p=0.003	r=-0.092 p=0.058	r=0.065 p=0.185	r=0.049 p=0.317	r=0.313 p=4x10^-11^	r=0.279 p=5x10^-9^	r=0.168 p=0.002	r=0.239 p=6x10^-7^	r=0.16 p=0.003	r=0.198 p=2x10^-4^	r=0.272 p=3x10^-7^	r==0.231 p=2x10^-5^	r=0.230 p=2x10^-5^	r=0.108 p=0.026	r=0.268 p=2x10^-8^				
**Angiogenin**	r=0.129 p=0.008	r=0.023 p=0.635	r=0.199 p=4E-05	r=0.165 p=0.001	r=-0.004 p=0.932	r=0.158 p=0.001	r=0.092 p=0.058	r=0.161 p=0.001	r=0.108 p=0.045	r=0.262 p=5x10^-8^	r=0.002 p=0.966	r=0.066 p=0.22	r=0.178 p=0.001	r=0.122 p=0.024	r=0.158 p=0.003	r=0.396 p=3x10^-17^					
**ICAM- 1**	r=-0.017 p=0.731	r=0.119 p=0.014	r=0.113 p=0.02	r=0.091 p=0.061	r=0.122 p=0.013	r=0.073 p=0.133	r=0.001 p=0.976	r=0.313 p=5x10^-11^	r=0.044 p=0.412	r=0.09 p=0.065	r=-0.082 p=0.131	r=-0.04 p=0.463	r=0.157 p=0.003	r=0.114 p=0.035	r=0.089 p=0.099						
**IL-1β**	r=-0.014 p=0.802	r=-0.091 p=0.094	r=0.077 p=0.157	r=0.19 p=4E-04	r=0.108 p=0.048	r=0.159 p=0.003	r=-0.056 p=0.3	r=-0.022 p=0.691	r=0.190 p=4x10^-4^	r=0.192 p=3x10^-4^	r=0.357 p=9x10^-12^	r=0.405 p=5x10^-15^	r=0.487 p=7x10^-22^	r=0.507 p=7x10^-24^							
**IL-4**	r=-0.013 p=0.815	r=-0.06 p=0.271	r=-0.009 p=0.869	r=0.013 p=0.817	r=0.116 p=0.035	r=0.104 p=0.055	r=-0.024 p=0.655	r=-0.006 p=0.907	r=0.19 p=4x10^-4^	r=0.043 p=0.426	r=0.386 p=1x10^-13^	r=0.289 p=5x10^-8^	r=0.484 p=10^-21^								
**IL-2**	r=0.007 p=0.89	r=-0.074 p=0.169	r=-0.007 p=0.892	r=0.03 p=0.573	r=0.008 p=0.891	r=0.049 p=0.362	r=0.095 p=0.077	r=-0.085 p=0.114	r=0.317 p=2x10^-9^	r=0.142 p=0.008	r=0.407 p=4x10^-15^	r=0.245 p=4x10^-6^									
**IL-12p70**	r=-0.071 p=0.191	r=-0.114 p=0.034	r=0.062 p=0.255	r=0.052 p=0.336	r=-0.039 p=0.473	r=0.12 p=0.027	r=-0.026 p=0.637	r=-0.084 p=0.122	r=0.22 p=4x10^-5^	r=0.184 p=0.001	r=0.373 p=8E-13										
**TNF**	r=0.006 p=0.915	r=-0.041 p=0.452	r=0.015 p=0.78	r=-0.048 p=0.37	r=0.011 p=0.836	r=-0.013 p=0.805	r=-0.062 p=0.254	r=-0.095 p=0.079	r=0.173 p=0.001	r=0.036 p=0.506											
**Granzyme A**	r=0.057 p=0.242	r=0.0002 p=0.996	r=0.285 p=2E-09	r=0.191 p=8E-05	r=-0.089 p=0.07	r=0.143 p=0.003	r=0.313 p=4x10^-11^	r=0.107 p=0.029	r=0.132 p=0.014												
**IFN-γ**	r=-0.04 p=0.462	r=-0.11 p=0.041	r=0.003 p=0.958	r=0.03 p=0.585	r=0.023 p=0.678	r=0.289 p=5x10^-8^	r=0.093 p=0.086	r=-0.001 p=0.985													
**TNF-RI**	r=0.14 p=0.004	r=0.178 p=2x10^-4^	r=0.226 p=3x10^-6^	r=0.114 p=0.02	r=0.222 p=5x10^-6^	r=0.068 p=0.164	r=0.183 p=2x10^-4^														
**IP-10**	r=0.049 p=0.315	r=-0.021 p=0.671	r=0.039 p=0.428	r=-0.192 p=7x10^-5^	r=-0.183 p=2x10^-4^	r=0.067 p=0.171															
**IL-17F**	r=-0.03 p=0.538	r=-0.134 p=0.006	r=0.104 p=0.032	r=0.037 p=0.45	r=-0.015 p=0.754																
**NLR**	r=0.025 p=0.618	r=0.077 p=0.116	r=0.123 p=0.012	r=0.259 p=9x10^-8^																	
**G-CSF**	r=0.163 p=0.001	r=0.088 p=0.071	r=0.254 p=10^-7^																		
**VEGF**	r=0.352 p=9x10^-14^	r=0.352 p=8x10^-14^																			
**CCL5**	r=0.37 p=4E-15																				

Weak, but significant correlations (|r|<0.3) were highlighted in light green, while stronger correlations were highlighted in deeper green. Markers with multiple (>1) stronger correlations with each other have been highlighted in light blue. Results with p<0.05, but FDR >0.1 are highlighted in grey.

IL, interleukin; IFN-γ, interferon gamma; TNF, tumour necrosis factor; ICAM-1, intercellular adhesion molecule 1; IP-10, interferon-gamma induced protein 10; VEGF, vascular endothelial growth factor; angiogenin, sCD40L, soluble CD40 ligand; G-CSF, granulocyte-colony stimulating factor; CCL5, CC motif chemokine ligand 5; TNF-RI, soluble TNF-receptor I.

Furthermore, the correlations between baseline serum cytokine levels and clinical characteristics are summarized in **Table 4**. IL-6 levels were higher in men (FC=4, p=0.003), while IP-10 showed a moderate positive correlation with age (r=0.323, p=4x10^-5^). All other associations noted were either non-significant or weak (|r|<0.3). In particular, no association was found between the serologic CMV status of patients and serum cytokine levels.

**Table 4:** Associations between blood cytokine levels and patient characteristics.

	sCD40L	CCL5	VEGF	G-CSF	NLR	IL-17F	IP-10	TNF-RI	IFN-γ	Gran-zyme A	TNF	IL-12p70	IL-2	IL-4	IL-1β	ICAM- 1	Angio-genin	IL-6	IL-5	IL-10	IL-8
**sex (m/w)**	FC=0.8 p=0.06	FC=0.9 p=0.20	FC=1.1 p=0.13	FC=3 p=0.49	FC=1.2 p=0.24	FC>10 p=0.11	FC=1.1 p=0.91	FC=1.1 p=0.14	FC>10 p=0.25	FC=2 p=0.29	FC=8 p=0.70	FC=10 p=0.98	FC=69 p=0.94	FC=143 p=0.89	FC=13 p=0.37	FC=4 p=0.78	FC=0.5 p=0.76	FC=4 p=0.003	FC=22 p=0.78	FC=11 p=0.75	FC=3 p=0.11
**age**	r=-0.25 p=0.002	r=-0.21 p=0.009	r=-0.01 p=0.884	r=-0.13 p=0.11	r=-0.11 p=0.11	r=0.10 p=0.202	r=0.32 p=4x10^-5^	r=0.18 p=0.029	r=0.07 p=0.487	r=0.12 p=0.124	r=0.01 p=0.931	r=0.08 p=0.393	r=0.01 p=0.956	r=-0.02 p=0.82	r=-0.19 p=0.035	r=0.153 p=0.058	r=-0.01 p=0.884	r=0.13 p=0.12	r=0.09 p=0.365	r=0.01 p=0.97	r=0.08 p=0.341
**smokers/** **non-smokers**	FC=0.9 p=0.71	FC=0.9 p=0.41	FC=1.8 p=0.03	FC=3 p=0.39	FC=1.1 p=0.70	FC=6 p=0.43	FC=1.0 p=0.16	FC=0.8 p=0.40	FC>10 p=0.57	FC=1.7 p=0.64	FC=98 p=0.48	FC=45 p=0.22	FC>10 p=0.11	FC=530 p=0.81	FC=196 p=0.75	FC=2 p=0.64	FC=1.6 p=0.42	FC=1.6 p=0.99	FC=18 p=0.55	FC=9 p=0.29	FC=1.9 p=0.50
**CMV** **pos/neg**	FC=1.1 p=0.54	FC=0.98 p=0.45	FC=0.8 p=0.79	FC=0.2 p=0.46	FC=1 p=0.25	FC<0.1 p=0.16	FC=1.3 p=0.47	FC=1.3 p=0.75	FC<0.1 p=0.17	FC=0.5 p=0.59	FC=0.1 p=0.38	FC=0.1 p=0.40	FC<0.1 p=0.81	FC=0.1 p=0.73	FC<0.1 p=0.26	FC=5 p=0.61	FC=0.9 p=0.72	FC=0.8 p=0.59	FC<0.1 p=0.19	FC<0.1 p=0.85	FC=0.5 p=0.59
**PD-L1 TPS** **(50+/1-49/0)**	r=-0.03 p=0.68	r=-0.20 p=0.014	r=0.109 p=0.178	r=-0.11 p=0.16	r=-0.13 p=0.11	r=0.4 p=0.65	r=0.26 p=0.001	r=-0.04 p=0.59	r=0.07 p=0.44	r=0.10 p=0.20	r=0.08 p=0.38	r=0.07 p=0.47	r=0.06 p=0.53	r=0.13 p=0.18	r=0.06 p=0.55	r=-0.19 p=0.02	r=0.03 p=0.67	r=0.25 p=0.002	r=-0.08 p=0.42	r=0.05 p=0.60	r=0.05 p=0.51
**No. of meta-** **static sites**	r=-0.01 p=0.91	r=-0.11 p=0.19	r=0.13 p=0.15	r=-0.05 p=0.59	r=0.07 p=0.41	r=0.04 p=0.69	r=0.04 p=0.63	r=0.23 p=0.006	r=0.05 p=0.62	r=0.04 p=0.66	r=0.06 p=0.53	r=-0.01 p=0.92	r=-0.02 p=0.83	r=0.08 p=0.42	r=-0.05 p=0.60	r=0.06 p=0.51	r=0.03 p=0.73	r=0.23 p=0.007	r=-0.12 p=0.23	r=0.07 p=0.47	r=0.20 p=0.02

Weak, but significant correlations (|r|<0.3 or FC=0.5-2) were highlighted in light green, while stronger correlations were highlighted in deeper green. Results with p<0.05, but FDR >0.1 are highlighted in grey.

IL, interleukin; IFN-γ, interferon gamma; TNF, tumour necrosis factor; ICAM-1, intercellular adhesion molecule 1; IP-10, interferon-gamma induced protein 10; VEGF, vascular endothelial growth factor; sCD40L, soluble CD40 ligand; G-CSF, granulocyte-colony stimulating factor; CCL5, CC motif chemokine ligand 5; TNF-RI, soluble TNF-receptor I; PD-L1, programmed cell death protein ligand 1; TPS, tumor proportion score.

## Discussion

Main objective of this study was to characterize the potential clinical utility of serum cytokine concentrations in IO-treated NSCLC. One prominent finding was the profoundly altered cytokine profile of newly diagnosed NSCLC patients compared to age-matched healthy controls (**Table 2** and **Figure 2**). Several interrelated (**Table 4**) mediators were significantly elevated, including the proinflammatory cytokines IL-6, IL-8, G-CSF and the proinflammatory chemokine CCL5 (25), the proinflammatory adhesion glycoprotein ICAM-1, soluble TNF-RI, which is increased in inflammatory states to curb the bioactivity of TNF (26, 27), the anti-inflammatory IL-10 (28), as well as the proangiogenic VEGF (29). These results illustrate the systemic inflammation and immune dysregulation present in metastatic NSCLC, which explains several disease manifestations and offers specific therapeutic vulnerabilities. For example, the increased NLR in the blood of these patients is facilitated by elevated levels of various interleukins and G-CSF, which stimulate granulopoiesis (30), ICAM-1 expression is induced by inflammation and associated with worse prognosis in NSCLC and other cancers likely by facilitating the metastatic cascade (31), while the therapeutic relevance of elevated VEGF in the circulation is reflected by the success of bevacizumab and other angiogenesis inhibitors in the treatment of metastatic NSCLC (32). Of note, association of blood cytokine levels with the tumor PD-L1 expression were generally absent or very weak (**Table 4**), so that these parameters capture different aspects of NSCLC immunobiology. For example, alterations of the lung and gut microbiome were associated with increased levels of several inflammatory serum cytokines, including IL-6 and TNF-α, in preclinical models of lung cancer, which could in part be remedied by the administration of probiotics (33, 34). At the same time, the association of IP-10 and CCL-5 with PD-L1 TPS (**Table 3**) probably explain why these were elevated only in 1L-IO (with higher PD-L1 expression ≥ 50%) or ICT patients (with lower average PD-L1 expression), respectively (**Table 2**).

In contrast, serum cytokine changes under treatment were subtle. Consistent was only a decrease in angiogenin, also known as ribonuclease 5, a small, 123 amino acid protein that stimulates angiogenesis alongside several other pleotropic effects (35). Recently it has been reported, that increased serum angiogenin correlated with dynamic contrast-enhanced MR-PET parameters in NSCLC patients, which were improved under anti-angiogenic therapy and linked with OS (36). However, in our study the change in angiogenin under immunotherapy was neither accompanied by a decrease in circulating VEGF nor associated with OS (**Table 2**), therefore its significance remains unclear. From a clinical perspective, an important conclusion from the results of this study is that serial serum cytokine measurements are not suitable for disease monitoring, since there were minimal changes under treatment in our cohort (**Figures 3A, B**). This is in contrast to longitudinal ctDNA assays, which have demonstrated potential clinical utility in the context of both immunotherapy-treated (37) and oncogene-driven disease (38), based on the strong association of higher tumor mutation levels in the blood under therapy with refractory disease and shorter PFS (39). In particular, serum cytokines are obviously not suitable for early detection of treatment failure, since no consistent changes accompanied PD in our patients, contrary to ctDNA-based liquid biopsies, which can reveal emergence of novel mutations of increases in allelic frequencies of preexisting variants as a sensitive marker of PD several months earlier than radiologic tumor growth according to recent pivotal studies (40). Moreover, dynamic changes of cytokine levels after 1 and 4 treatment cycles were not associated with immunotherapeutic efficacy (LR *vs.* RP, **Table 2**), while dynamic changes of the NLR after 4 cycles (12 weeks) correlated with clinical outcome, as has also been observed by other investigators (41). Besides low sensitivity, as demonstrated by the current study, another problem of disease monitoring using serum cytokines would be susceptibility to external influences by factors unrelated to the tumor remission status, like use of steroids and concomitant infection (42, 43). The lack of association between serum cytokine concentrations in NSCLC and the serologic CMV status (**Table 4**), itself linked to mild chronic immune activation and immunosenescence (44), also reflects the inability of cytokines to capture subtle systemic changes of the adaptive immunity, as those expected to occur longitudinally under PD-(L)1 blockade.

Another important question is whether baseline cytokine levels could be used for improved prediction of immunotherapy benefit. In general, few differences in the blood levels of analyzed cytokines between patients with LR *vs.* RP were noted, collectively suggesting an association between lower levels of inflammatory markers, such as IL-6, IL-8, IP-10, TNF-RI and the NRL, with better immunotherapy outcome (**Table 2**). Similar observations have recently been reported by other investigators, as well, for example lower IL-6 and IL-8 levels at baseline as well as after 1 cycle of treatment were strongly linked to longer survival under immunotherapy in patients with lung cancer and melanoma from a prospective multicenter study in Italy (45). Based on the results of the current study, low levels of TNF-RI appear to be a particularly promising marker for several reasons: first, for patients receiving PD-(L)1 inhibitor monotherapy, the TNF-RI differences between LR *vs.* RP were more pronounced than those observed for the established marker NLR (FC=0.5 with p=0.003 *vs.* FC=0.8 with p=0.024, **Table 2**) (46); second, TNF-RI retained prognostic utility also for patients treated with ICT (FC=0.8 with p=0.006, **Table 2** and **Figure 4**), which is a major unmet need, because the NLR, ALI, PD-L1 and other biomarkers of PD-(L)1 monotherapy become useless, when additional chemotherapy is administered (19); third, blood TNF-RI levels showed no correlation with tissue PD-L1 expression (**Table 4**), which means that it represents an independent biomarker; finally, the signal of TNF-RI observed in this study appears to be stronger than that of several other inflammatory biomarkers described in the literature, like the blood levels of IL-6 (47), IL-8 (48), IP-10 (49), ICAM-1 (50) and VEGF (51). Actually, all these molecules showed significantly increased levels at baseline in our patients, similar to TNF-RI, but lower levels of TNF-RI could much better discriminate LR *vs.* RP patients (**Table 2** and **Supplementary Table 4**).

Remarkable was also the association of several serum cytokines, in particular increased angiogenin and IL-1β, but also IL-5, IL-8, IL-10, IL-12p70, and granzyme A, or decreased G-CSF at baseline, with the subsequent development of irAE (**Figures 4E, F**). Higher baseline IL-1β, IL-10 and IL-12p70 serum concentrations in NSCLC patients who later developed rheumatic irAE have been independently confirmed in a different patient cohort (personal communication with KB and MMSC). Such an association between preexisting systemic inflammation and autoimmunity has also been observed in other tumor types, like indolent B-cell lymphomas (52) and malignant thymoma (53). These results corroborate previous reports of a higher propensity for the development of irAE in IO-treated cancer patients with higher blood IL-10 (54) and other inflammatory mediators (55). Of note, irAE-related cytokines did not include TNF-RI and IL-6, whose lower levels were associated with longer survival in this study (**Table 2**), so that a complex protein panel may be able to independently predict both IO efficacy and IO toxicity. The association of baseline IL-1β levels with irAE is particularly interesting, because it had not been reported in NSCLC patients before, and because the IL-1β inhibitor canakinumab and other IL-1 drugs are widely, in part off-label, used to treat a variety of mainly autoinflammatory disorders in rheumatology (56). There is also evidence suggesting anticancer activity of canakinumab, for example its use was associated with a reduced incidence of lung cancer in the phase 3 CANTOS study (57), so that phase 3 trials of this drug in combination with chemotherapy for the treatment of NSCLC in various stages are ongoing (58). The findings of this study suggest that these drugs could potentially be useful in the treatment or even prevention of irAE, as well.

Main advantages of this work are the relatively large number of cytokines based on a preceding systematic literature review, the relatively large number of patients in several dedicated cohorts, *i.e.* first-line PD-(L)1 monotherapy, ICT, or PD-(L)1 monotherapy in subsequent lines, the prospective longitudinal sample collection at defined uniform time-points, the simultaneous consideration of IO efficacy and toxicity, and the rigorous statistical testing including correction for multiple comparisons and multivariable testing. Main limitations are the smaller number of available samples under treatment and at disease progression, as well as the inability to exclude potential confounders. Therefore, the results will need to be validated in future studies, which might pave the way for building a complex score based on several cytokines and data mining analysis (59). Other emerging approaches to refine patient stratification are measurement of circulating tumor cells, ctDNA, miRNA, blood exosomes, gene expression profiling, or analysis of the T-cell receptor (TCR) repertoire (18, 37, 60–64).

## Conclusion

In conclusion, this study could demonstrate that several altered serum cytokines in patients with advanced NSCLC could be exploited in order to predict efficacy and toxicity of PD-(L)1 monotherapy or ICT more accurately, but they are not suitable for longitudinal disease monitoring and early detection of tumor escape.

## Data availability statement

The raw data supporting the conclusions of this article will be made available by the authors, without undue reservation.

## Ethics statement

The studies involving human participants were reviewed and approved by Ethics committee of Heidelberg University (S-579/2019). All patients and healthy controls provided written informed consent to participate in this study.

## Author contributions

HaS: Conceptualization, Methodology, Investigation; Data curation, Formal analysis, Visualization, Writing - Original Draft, Writing - Review and Editing; FL: Methodology, Investigation; Data curation, Formal analysis, Visualization, Writing - Review and Editing; ME: Investigation, Data curation, Validation, Writing - Review and Editing; LD: Investigation, Data curation, Validation, Writing - Review and Editing; LG: Investigation, Data curation, Validation, Writing - Review and Editing; KB: Data curation, Validation, Writing – Review and Editing; MS-C: Data curation, Validation, Writing – Review and Editing; AA: Validation, Writing – Review and Editing; FJ: Validation, Writing - Review and Editing; FE: Validation, Writing - Review and Editing; DK: Data curation, Validation, Writing – Review and Editing; MS: Investigation, Writing - Review and Editing; SL: Data curation, Validation, Writing - Review and Editing; SK: Investigation; Data curation, Formal analysis, Writing - Review and Editing; PS: Investigation, Data curation, Validation, Writing - Review and Editing; AS: Validation, Supervision, Writing - Review and Editing; HoS: Investigation, Data curation, Validation, Writing - Review and Editing; MT: Conceptualization, Methodology, Validation, Supervision, Funding acquisition, Writing - Review and Editing; PC: Conceptualization, Methodology, Investigation; Data curation, Formal analysis, Visualization, Supervision, Project administration; Writing - Original Draft, Writing - Review and Editing. All authors contributed to the article and approved the submitted version.

## Funding

This work was supported by the German Center for Lung Research (DZL 3.0).

## Acknowledgments

We would like to thank Simone Kuder for help with the data collection, Ingrid Heinzmann-Groth for help with the collection of patient samples, as well as Tamara Hedinger, Simone Karcher-Bausch and Simone Butz for expert technical assistance.

## Conflict of interest

Author KB received consultancy and/or speaker fees and/or travel reimbursements from Abbvie, Bristol Myers Squibb BMS, Gilead/Galapagos, Janssen, Merck Sharp & Dohme MSD, Mundipharma, Novartis, Pfizer, Roche, Viatris, UCB, as well as scientific support from the Medical Faculty of University of Heidelberg, Rheumaliga Baden-Württemberg e.V., AbbVie, and Novartis. Author FE received personal fees from Roche and BMS; DK: advisory board and speaker’s honoraria from AstraZeneca, BMS, Pfizer. Author AS received advisory board honoraria from BMS, AstraZeneca, ThermoFisher, Novartis, speaker’s honoraria from BMS, Illumina, AstraZeneca, Novartis, ThermoFisher, MSD, Roche, and research funding from Chugai. Author HoS received research grants and personal fees from Roche Sequencing Solutions, outside the submitted work. Author MT received advisory board honoraria from Novartis, Lilly, BMS, MSD, Roche, Celgene, Takeda, AbbVie, Boehringer, speaker’s honoraria from Lilly, MSD, Takeda, research funding from AstraZeneca, BMS, Celgene, Novartis, Roche and travel grants from BMS, MSD, Novartis, Boehringer. Author PC received research funding from Amgen, AstraZeneca, Merck, Novartis, Roche, Takeda, and advisory board/lecture fees from AstraZeneca, Boehringer Ingelheim, Chugai, Daiichi Sankyo, Gilead, Novartis, Pfizer, Roche, Takeda.

The remaining authors declare that the research was conducted in the absence of any commercial or financial relationships that could be construed as a potential conflict of interest.

## Publisher’s note

All claims expressed in this article are solely those of the authors and do not necessarily represent those of their affiliated organizations, or those of the publisher, the editors and the reviewers. Any product that may be evaluated in this article, or claim that may be made by its manufacturer, is not guaranteed or endorsed by the publisher.
