# Supplementary material for: Serum cytokines predict efficacy and toxicity, but are not useful for disease monitoring in lung cancer treated with PD-(L)1 inhibitors
Source: Front Oncol. 2022 Oct 31;12:1010660. doi: 10.3389/fonc.2022.1010660 (PMC9662790; doi:10.3389/fonc.2022.1010660)

Supplementary Material

Supplementary Tables:

Supplementary Table 1: published evidence about serum biomarkers of immunotherapy in non-small-cell lung cancer.

Published evidence about the potential association of 100 serum markers with the clinical outcome of non-small-cell lung cancer patients receiving PD-(L)1 inhibitors was collected from 624 original publications identified in Pubmed as described in the Methods section (search terms ((predictive biomarker[Title/Abstract]) AND ((NSCLC[Title/Abstract]) OR (lung cancer[Title/Abstract]))). For each of the serum markers listed below, each character of the string in the "evidence" column represents one study, while the study results are coded according to the following rule: 1 = association of the marker with PD-(L)1 inhibitor efficacy; 3 = association of the marker with the development of immune-related adverse events (toxicity); 5 = association of the marker with both efficacy and toxicity; 0 = no associated of the marker with efficacy or toxicity. Markers analyzed in the current study are highlighted in green, and their selection was based on both the previously published evidence and technical feasibility of parallel measurement (n=16). In addition, four promising markers with different published evidence were explored: two with data under (chemo-)immunotherapy for melanoma, but not NSCLC: angiogenin (PMID 17762972) and granzyme A (PMID 33746582); one with data for chemotherapy, but not immunotherapy in NSCLC: ICAM-1 (PMID 21550560, 23884579, 32853940, 25202042); and one with preclinical only data about relationship with anti-tumor immunity: IL-17F (PMID 32227296, 20635888).

Serum marker	evidence	Serum marker	evidence
IL-8 (CXCL8)	1111110000	IL-13	100
IP-10 (CXCL10)	11130	IL-1RA	100
RANTES (CCL5)	11300	BMP-9	30
IFN-γ	1300000000	CXCL11 (I-TAC)	30
G-CSF	3010	Leptin	30
IL-10	100000000	IL-12P40	30
IL-2	11300000	IL-16	30
TNF-α	11300000	CD137 (4-1-BB, TNFRSF9)	10
IL-6	11100000	FGF	10
IL-4	1100000	Follistatin	10
IL-5	1000	Granzyme B	10
VEGF	1000	IL-18	10
IL-1β	100000	sGITR	10
sTNFRI	1	CXCL19	3
IL-12P70	0	sPD-L2	3
sCD40L	0	Perforin	1
MCP-1 (CCL2)	110000	sIDO	1
sPD-L1	110	Angiopoietin-2	0
Eotaxin (CCL11)	100000	b-NGF	0
MIP-1α (CCL3)	100000	CCL21 (6kine)	0
GRO (CXCL1)	50000	CTACK (CCL27)	0
MIP-1β (CCL4)	10000	CXCL5 (ENA-78)	0
IL-7	1000	CXCL13 (BCA-1)	0
CXCL9 (MIG)	300	EGF	0
MCP-3 (CCL7)	300	Endoglin	0

HGF	100	Endothelin-1	0
IL-12(IL23p40)	100	Eotaxin-2 (CCL24)	0
Eotaxin-3 (CCL26)	0	GRO-b (CXCL2)	0
Fas-Ligand	0	HB-EGF	0
FGF-1	0	IFN-α2	0
FGF-2	0	IL-15	0
Flt-3L	0	IL-17	0
Fractalkine (CXCL1)	0	IL-17A/17	0
GCP-2 (CXCL6)	0	IL-1α	0
GM-CSF	0	IL-2RA	0
I-309 (CCL1)	0	IL-3	0
IL-33	0	PLGF	0
IL-9	0	SCGF-b	0
LIF	0	SCYB16 (CXCL16)	0
M-CSF	0	SDF-1a (CXCL12)	0
MCP-2 (CCL8)	0	TARC (CCL17)	0
MCP-4 (CCL13)	0	TGF-α	0
MDC (CCL22)	0	TGF-β1	0
MIF	0	TGF-β2	0
MIP-1δ (CCL15)	0	TGF-β3	0
MIP-3α (CCL20)	0	TNF-β	0
MIP-3β (CCL19)	0	TRAIL	0
MPIF-1 (CCL23)	0	VEGF-A	0
PDGF-AA	0	VEGF-C	0
PDGF-AB/BB	0	VEGF-D	0

Supplementary Table 2: Cytokine assay characteristics

**Cytokine**	**Vendor**	**Sensitivity**	**Range standard curve**	**Limit of detection**
IL-1β	BD	E	274-200000 fg/ml	48.4 fg/ml
IL-2	BD	E	274-200000 fg/ml	88.9 fg/ml
IL-4	BD	E	274-200000 fg/ml	144.4 fg/ml
IL-5	BD	E	274-200000 fg/ml	67.8 fg/ml
IL-6	BD	E	274-200000 fg/ml	68.4 fg/ml
IL-8	BD	E	274-200000 fg/ml	69.9 fg/ml
IL-10	BD	E	274-200000 fg/ml	13.7 fg/ml
IL-12p70	BD	E	274-200000 fg/ml	12.6 fg/ml
IL-17F	BD	S	10-2500 pg/ml	2.9 pg/ml
IFN-γ	BD	E	274-200000 fg/ml	14.8 fg/ml
TNF	BD	E	274-200000 fg/ml	67.3 fg/ml
ICAM-1	BD	S	40-10000 pg/ml	25.7 pg/ml
IP-10	BD	S	10-2500 pg/ml	0.5 pg/ml
VEGF	BD	S	10-2500 pg/ml	4.5 pg/ml
angiogenin	BD	S	10-2500 pg/ml	4.6 pg/ml
sCD40L	BD	S	10-2500 pg/ml	2.3 pg/ml
G-CSF	BD	S	10-2500 pg/ml	1.6 pg/ml
CCL5	BD	S	10-2500 pg/ml	0.002 pg/ml
Granzyme a	BD	S	40-10000 pg/ml	3.7 pg/ml
TNF-RI	BD	S	40-10000 pg/ml	5.2 pg/ml

S: standard sensitivity; E: enhanced sensitivity

Supplementary Table 3: Patient characteristics according to IO efficacy

	1L-IO		ICT		2L-IO		>2L-IO	
	**RP** (n = 19)	**LR** (n = 27)	**RP** (n = 34)	**LR** (n = 74)	**RP** (n = 19)	**LR** (n = 15)	**RP** (n = 12)	**LR** (n = 4)
**Samples (n)**								
**1C**	2	9	8	19	0	0	0	0
**4C**	5	23	4	63	7	15	0	4
**PD**	3	5	16	11	5	6	1	1
**Age (mean, range)**	72 (58-87)	66 (48-83)	65 (49-87)	63 (37-81)	64 (51-78)	64 (48-78)	61 (50-71)	65 (60-71)
**Sex (n, %)**								
**Female**	5 (26%)	7 (26%)	8 (24%)	31 (41,9%)	7 (37%)	7 (47%)	8 (67%)	3 (75%)
**Male**	14 (74%)	20 (74%)	26 (76%)	43 (58,1%)	12 (63%)	8 (53%)	4 (33%)	1 (25%)
**Smoker s(n, %)**								
**never**	2 (11%)	2 (7%)	5 (15%)	6 (8%)	0	0	0	1 (25%)
**former**	13 (68%)	11 (41%)	19 (56%)	33 (45%)	9 (47%)	11 (73%)	7 (58%)	1 (25%)
**current**	4 (21%)	14 (52%)	10 (29%)	35 (47%)	10 (53%)	4 (27%)	5 (42%)	2 (50%)
**ECOG (n, %)**								
**0**	8 (42%)	11 (41%)	14 (41%)	34 (46%)	7 (37%)	6 (40%)	3 (25%)	3 (75%)
**1**	10 (53%)	16 (59%)	19 (56%)	39 (53%)	11 (58%)	8 (53%)	5 (42%)	1 (25%)
**≥ 2**	1 (5%)	0	1 (3%)	1 (1%)	1 (5%)	1 (7%)	4 (33%)	0
**PD-L1 TPS (n, %)**								
**<1**	1 (5%)	0	13 (38%)	20 (27%)	6 (32%)	1 (7%)	5 (42%)	1 (25%)
**1-49**	6 (32%)	4 (15%)	15 (44%)	30 (41%)	12 (63%)	9 (60%)	4 (33%)	3 (75%)
**≥50**	12 (63%)	23 (85%)	6 (18%)	23 (32%)	1 (5%)	5 (33%)	3 (25%)	0
**Histology (n, %)**								
**ADC**	10 (53%)	16 (59%)	24 (70%)	59 (80%)	10 (53%)	10 (66%)	9 (75%)	4 (100%)
**SCC**	5 (26%)	9 (33%)	5 (15%)	9 (12%)	7 (37%)	4 (27%)	1 (8%)	0
**Other NSCLC**	4 (21%)	2 (7%)	5 (15%)	6 (8%)	2 (10%)	1 (7%)	2 (17%)	0
**Immunotherapy**								
**anti-PD-1**	17 (89%)	27 (100%)	33 (97%)	74 (100%)	12 (63%)	11 (73%)	9 (75%)	3 (75%)
**anti- PD-L1**	2 (11%)	0	1 (3%)	0	7 (37%)	4 (27%)	3 (25%)	1 (25%)
**irAE (n, %)**								
**Yes**	3 (16%)	11 (41%)	4 (12%)	15 (20%)	4 (21%)	2 (13%)	2 (17%)	2 (50%)
**No**	16 (84%)	16 (59%)	30 (88%)	59 (80%)	15 (79%)	13 (87%)	10 (83%)	2 (50%)

NSCLC: non-small-cell lung cancer; ICT: immunochemotherapy; 1L-IO: patients receiving PD-(L)1 inhibitors as monotherapy in the first line; 2+L-IO: patients receiving PD-(L)1 inhibitors as monotherapy in the second-or-subsequent lines; RP: rapid progression (progression-free survival (PFS) < 120 days); LR: long-time response (PFS > 200 days); BL: baseline; C1: sample after 1 cycle of treatment; C4: sample after 4 cycles of treatment; PD: progressive disease; ECOG: Eastern Cooperative Oncology Group; PD-L1: programmed cell death protein ligand 1; TPS: tumor proportion score; ADC: adenocarcinoma; SCC: squamous-cell carcinoma, NSCLC: non-small-cell lung cancer; irAE: immune-related adverse events; 1L: first line.

[1] PD-1-inhibitors: nivolumab, pembrolizumab; PD-L1-inhibitors: atezolizumab, durvalumab.

Supplementary Table 4: Association of serum cytokines with IO efficacy and irAE in patient groups

Marker	LR vs. RP ICT				ICT irAE	LR vs. RP 1L-IO				1L-IO irAE	LR vs. RP 2+L-IO			2+L-IO irAE
	baseline	under treatment			baseline	baseline	under treatment			baseline	baseline	under treatment		baseline
	(n=108)	C1<br>(n=27)	C4<br>(n=83)	PD<br>(n=27)	vs. ctrl<br>(n=108)	(n=46)	C1<br>(n=11)	C4<br>(n=28)	PD<br>(n=7)	vs. ctrl<br>(n=46)	(n=50)	C4<br>(n=27)	PD<br>(n=13)	vs. ctrl<br>(n=50)
IL-1β	FC=3<br>p=0.689	FC=1<br>p=1	FC=1.4<br>p=0.073	FC<0.1<br>p=0.786	FC=7<br>p=0.009	FC=0.8<br>p=0.817	FC=1<br>p=	FC=3<br>p=0.268	FC=1<br>p=1	FC=0.1<br>p=0.617	FC=0.9<br>p=0.791	FC>10<br>p=0.359	FC>10<br>p=0.355	FC=1.2<br>p=0.436
IL-2	FC=2<br>p=0.898	FC>10<br>p=0.35	FC>10<br>p=0.638	FC<0.1<br>p=0.407	FC=4<br>p=0.773	FC=9<br>p=0.605	FC>10<br>p=0.637	FC>10<br>p=0.637	FC=1<br>p=1	FC=0.1<br>p=0.135	FC=0.8<br>p=0.224	FC=0.5<br>p=0.91	FC=1<br>p=1	FC=0.5<br>p=0.127
IL-4	FC=49<br>p=0.651	FC>10<br>p=0.516	FC>10<br>p=0.506	FC=0.7<br>p=0.344	FC=88<br>p=0.056	FC=0.5<br>p=0.908	FC>10<br>p=0.484	FC=1.8<br>p=0.476	FC>10<br>p=0.386	FC=1.7<br>p=0.685	FC=0.5<br>p=0.501	FC=0.2<br>p=0.118	FC=1.7<br>p=0.499	FC=5<br>p=0.125
IL-5	FC=3<br>p=0.271	FC<0.1<br>p=0.483	FC>10<br>p=0.53	FC<0.1<br>p=0.828	FC=4<br>p=0.011	FC=213<br>p=0.034	FC>10<br>p=0.484	FC>10<br>p=0.193	FC=1<br>p=1	FC=24<br>p=0.092	FC=4<br>p=0.558	FC=6<br>p=0.949	FC=1<br>p=1	FC=17<br>p=0.003
IL-6	FC=0.7<br>p=0.005	FC=0.1<br>p=0.492	FC<0.1<br>p=0.116	FC=0.5<br>p=0.198	FC=0.4<br>p=0.473	FC=0.6<br>p=0.631	FC=0.8<br>p=0.813	FC=0.9<br>p=0.927	FC=0.1<br>p=0.285	FC=1<br>p=0.267	FC=1<br>p=0.652	FC=2<br>p=0.466	FC=12<br>p=0.114	FC=0.7<br>p=0.865
IL-8	FC=0.7<br>p=0.016	FC=1.9<br>p=0.167	FC=0.5<br>p=0.843	FC=0.6<br>p=0.132	FC=1.6<br>p=0.028	FC=0.7<br>p=0.569	FC=1.7<br>p=0.723	FC=0.6<br>p=0.653	FC=1<br>p=0.881	FC=1.2<br>p=0.283	FC=0.7<br>p=0.197	FC=1.4<br>p=0.31	FC=4<br>p=0.317	FC=0.9<br>p=0.903
IL-10	FC=1<br>p=0.694	FC=0.7<br>p=0.499	FC=0.4<br>p=0.263	FC<0.1<br>p=0.029	FC=3<br>p=0.004	FC=2<br>p=0.829	FC>10<br>p=0.275	FC=7<br>p=0.966	FC=0.3<br>p=0.714	FC=5<br>p=0.091	FC=1<br>p=0.459	FC=1.5<br>p=0.829	FC=10<br>p=0.071	FC=1.7<br>p=0.173
IP-10	FC=0.8<br>p=0.037	FC=0.2<br>p=0.167	FC=0.3<br>p=0.223	FC=0.6<br>p=0.103	FC=0.8<br>p=0.166	FC=0.8<br>p=0.729	FC=1.5<br>p=0.48	FC=0.7<br>p=0.741	FC=0.6<br>p=0.456	FC=1.4<br>p=0.316	FC=0.7<br>p=0.43	FC=0.6<br>p=0.157	FC=1.4<br>p=0.253	FC=0.6<br>p=0.225
IL-12p70	FC=5<br>p=0.857	FC=15<br>p=0.884	FC>10<br>p=0.53	FC=1.7<br>p=0.588	FC=4<br>p=0.019	FC>10<br>p=0.056	FC>10<br>p=0.275	FC>10<br>p=0.392	FC=1<br>p=1	FC=9<br>p=0.726	FC=0.4<br>p=0.508	FC=0.6<br>p=0.364	FC=17<br>p=0.499	FC=9<br>p=0.014
IL-17F	FC=13<br>p=0.193	FC=1<br>p=1	FC=1<br>p=1	FC=1<br>p=1	FC<0.1<br>p=0.419	FC>10<br>p=0.402	FC=1<br>p=1	FC>10<br>p=0.502	FC=1<br>p=1	FC>10<br>p=0.131	FC>10<br>p=0.201	FC=1<br>p=1	FC=1<br>p=1	FC>10<br>p=0.046
CCL5	FC=1.1<br>p=0.171	FC=0.9<br>p=0.426	FC=0.8<br>p=0.328	FC=0.9<br>p=0.693	FC=0.9<br>p=0.24	FC=0.8<br>p=0.713	FC=1.1<br>p=0.814	FC=0.7<br>p=0.418	FC=1.2<br>p=0.456	FC=0.9<br>p=0.567	FC=1<br>p=0.834	FC=0.7<br>p=0.078	FC=0.8<br>p=0.317	FC=0.7<br>p=0.085
sCD40L	FC=1.1<br>p=0.721	FC=0.6<br>p=0.1	FC=1.1<br>p=0.832	FC=1.3<br>p=0.103	FC=1.2<br>p=0.631	FC=0.999<br>p=0.746	FC=1.9<br>p=0.346	FC=0.5<br>p=0.159	FC=1.1<br>p=0.297	FC=0.5<br>p=0.014	FC=0.7<br>p=0.047	FC=0.7<br>p=0.174	FC=1.2<br>p=0.886	FC=0.7<br>p=0.594
G-CSF	FC=1.5<br>p=0.344	FC=1.7<br>p=0.202	FC=0.8<br>p=0.491	FC=0.3<br>p=0.048	FC=0.7<br>p=0.007	FC=0.4<br>p=0.63	FC=0.7<br>p=0.346	FC=0.8<br>p=0.381	FC=1.7<br>p=0.18	FC=0.7<br>p=0.774	FC=0.8<br>p=0.96	FC=0.5<br>p=0.203	FC=0.7<br>p=0.431	FC=0.7<br>p=0.297
Granzyme A	FC=0.5<br>p=0.997	FC=1.9<br>p=0.08	FC=1.4<br>p=0.874	FC=0.2<br>p=0.627	FC=0.6<br>p=0.455	FC=1.3<br>p=0.188	FC=0.8<br>p=0.48	FC=2<br>p=0.126	FC=0.6<br>p=0.647	FC=1.6<br>p=0.037	FC=2<br>p=0.535	FC=0.9<br>p=0.37	FC=0.6<br>p=0.153	FC=1.3<br>p=0.01
ICAM-1	FC=2<br>p=0.776	FC=1.8<br>p=0.034	FC=24<br>p=0.711	FC=9<br>p=0.521	FC=0.1<br>p=0.75	FC=0.5<br>p=0.177	FC=0.2<br>p=0.059	FC=23<br>p=0.159	FC=8<br>p=0.456	FC=0.5<br>p=0.83	FC<0.1<br>p=0.689	FC<0.1<br>p=0.623	FC=2<br>p=0.01	FC=0.1<br>p=0.28
IFN-γ	FC>10<br>p=0.444	FC=1<br>p=1	FC=1<br>p=1	FC=1<br>p=1	FC<0.1<br>p=0.567	FC>10<br>p=0.505	FC=1<br>p=1	FC>10<br>p=0.637	FC=1<br>p=1	FC>10<br>p=0.181	FC>10<br>p=0.257	FC=1<br>p=1	FC=1<br>p=1	FC<0.1<br>p=0.5
TNF	FC=5<br>p=0.897	FC=1.2<br>p=0.811	FC=20<br>p=0.946	FC=0.5<br>p=0.53	FC=6<br>p=0.548	FC=8<br>p=0.567	FC>10<br>p=0.091	FC>10<br>p=0.193	FC=0.3<br>p=1	FC=0.1<br>p=0.023	FC=0.8<br>p=0.937	FC=0.2<br>p=1	FC=4<br>p=0.044	FC=1.5<br>p=0.933
TNF-RI	FC=0.8<br>p=0.006	FC=0.7<br>p=0.124	FC=0.3<br>p=0.053	FC=0.5<br>p=0.152	FC=0.7<br>p=0.062	FC=0.5<br>p=0.003	FC=1<br>p=1	FC=0.6<br>p=0.024	FC=0.4<br>p=0.025	FC=1.1<br>p=0.567	FC=0.8<br>p=0.153	FC=0.8<br>p=0.402	FC=0.9<br>p=0.475	FC=0.8<br>p=0.275
Angiogenin	FC=0.6<br>p=0.068	FC=1<br>p=0.524	FC=3<br>p=0.853	FC=0.3<br>p=0.693	FC=9<br>p=0.0002	FC=2<br>p=0.031	FC=1.5<br>p=0.48	FC=31<br>p=0.018	FC=1.4<br>p=0.297	FC=2<br>p=0.022	FC=3<br>p=0.084	FC=1.7<br>p=0.47	FC=0.7<br>p=0.116	FC=3<br>p=0.332
VEGF	FC=0.8<br>p=0.137	FC=0.8<br>p=0.137	FC=0.3<br>p=0.085	FC=0.7<br>p=0.49	FC=0.7<br>p=0.204	FC=0.8<br>p=0.631	FC=0.8<br>p=0.637	FC=1<br>p=0.976	FC=1.5<br>p=0.655	FC=0.8<br>p=0.445	FC=1<br>p=0.772	FC=1<br>p=0.47	FC=1<br>p=0.668	FC=1.3<br>p=0.357
NLR	FC=1.1<br>p=0.963	FC=0.9<br>p=0.873	FC=0.7<br>p=0.115	FC=0.6<br>p=0.2	FC=1.03<br>p=0.404	FC=0.8<br>p=0.024	FC=0.5<br>p=0.157	FC=0.5<br>p=0.029	FC=0.6<br>p=1	FC=0.6<br>p=0.126	FC=0.7<br>p=0.153	FC=0.6<br>p=0.022	FC=1.4<br>p=0.5	FC=1.1<br>p=0.96

Results with p<0.05 have been marked in green if passing and in gray if failing the false discovery rate <0.1 threshold. For abbreviations, please see Table 2.

Supplementary Table 5: Characteristics of immune-related adverse events (irAE) in study patients

	**1L-IO** (n=14)	**ICT** (n=19)	**2+L-IO** (n=10)
**irAE Grade (n, %)**			
**1**	0	5 (26%)	2 (20%)
**2**	3 (21%)	7 (37%)	5 (50%)
**3**	9 (64%)	7 (37%)	2 (20%)
**4**	2 (14%)	0	1 (10%)
**Affected organ system (n, %)**			
**Skin**	1 (7%)	0	3 (30%)
**Liver**	1 (7%)	1 (5%)	2 (20%)
**Colon**	6 (43%)	1 (5%)	1 (10%)
**Musculoskeletal**	3 (21%)	4 (21%)	0
**Lung**	1 (7%)	2 (11%)	2 (20%)
**Endocrine**	2 (14%)	6 (32%)	2 (20%)
**Heart**	0	2 (11%)	0
**Blood system**	0	1 (5%)	0
**Nervous system**	0	1 (5%)	0
**Kidney**	0	1 (5%)	0
**Treatment with steroids (n, %)**			
**Yes**	13 (93%)	14 (74%)	7 (70%)
**No**	1 (7%)	5 (26%)	3 (30%)

irAE: immune-related adverse event; ICT: immunochemotherapy; 1L-IO: patients receiving PD-(L)1 inhibitors as monotherapy in the first line; ctrl: age-matched heatlhy contros; 2+L-IO: patients receiving PD-(L)1 inhibitors as monotherapy in the second-or-subsequent lines

Supplementary Figures:

Supplementary Figure 1: Receiver operating characteristic curve for TNF-RI

Receiver operating characteristic (ROC) curve and Youden index analysis of the long-term responder (LR) vs. rapid progressor (RP) status of patients with metastatic NSCLC under first-line (chemo-)immunotherapy was used in order to derive an appropriate cut-off for the baseline serum TNF-RI concentration at the time of diagnosis. Area under the curve (AUC) was 0.703 (95% confidence interval 0.612-0.794), p=0.00003. The TNF-RI cut-off was 2139.7 pg/ml.

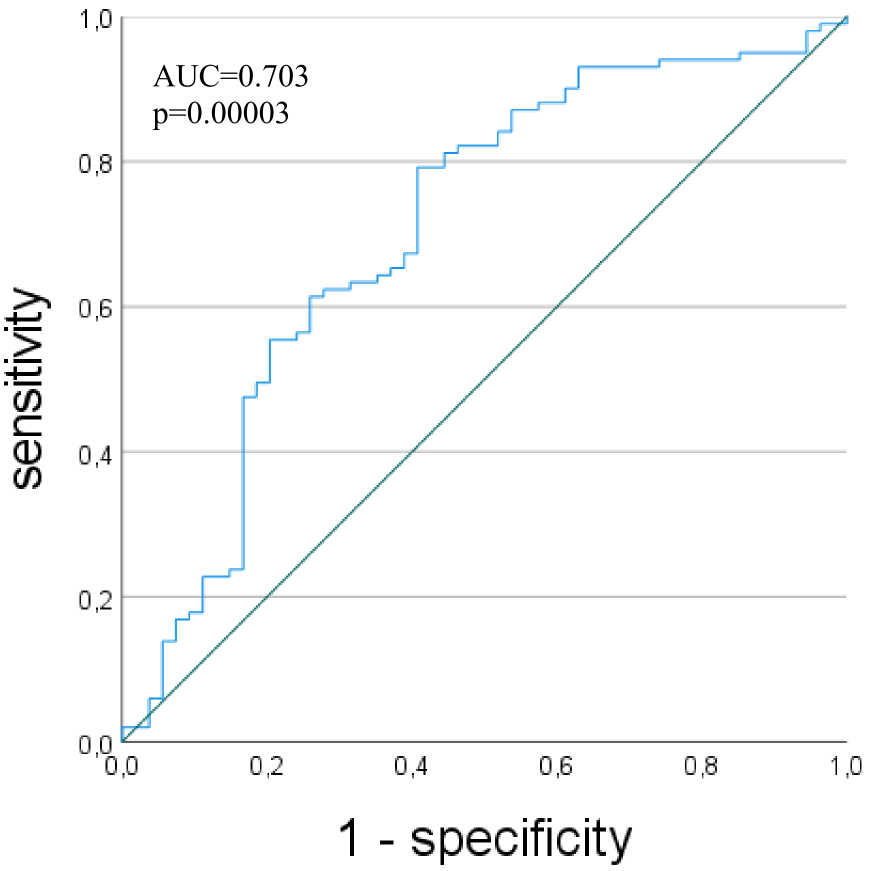

Supplement: Supplementary file 1 [file DataSheet_1.pdf]
